# Improvement in sleep duration was associated with higher cognitive function: a new association

**DOI:** 10.18632/aging.103948

**Published:** 2020-10-20

**Authors:** Jianian Hua, Hongpeng Sun, Yueping Shen

**Affiliations:** 1 Department of Neurology, The First Affiliated Hospital of Soochow University, Suzhou 215006, Jiangsu Province, PR China; 2 Medical College of Soochow University, Suzhou 215123, PR China; 3 Department of Child Health, School of Public Health, Medical College of Soochow University, Suzhou 215123, PR China; 4 Department of Epidemiology and Biostatistics, School of Public Health, Medical College of Soochow University, Suzhou 215123, PR China

**Keywords:** changes in sleep duration, cognitive function, cohort study, ageing, Chinese population

## Abstract

Objectives: Previous studies have indicated that improvement in sleep duration might correlate with better cognition. We aimed to examine the associations between changes in sleep duration and cognitive function.

Results: A change from short sleep duration (SSD) to moderate sleep duration (MSD) was associated with better global cognition scores (β=0.54, *P* <0.01). A change from SSD to long sleep duration (LSD) (β=-0.94, *P* <0.001) or a change from LSD to SSD (β=-1.38, *P* <0.01) was associated with lower global cognition. For individuals with MSD, a≥2 h increase (β=-0.89, *P* <0.001) or decrease (β=-0.70, *P* <0.001) in sleep duration was associated with lower global cognition.

Conclusions: For short sleepers, improvement in sleep duration correlated with better cognition. For long sleepers, there was no need to reduce sleep duration. Excessive changes or deviation from the moderate duration was associated with lower cognition.

Methods: A total of 10325 individuals aged 45 and older from the China Health and Retirement Longitudinal Study (CHARLS) were included. Self-reported nocturnal sleep duration and cognitive function were assessed in the three waves of CHARLS from 2011 to 2015. Cognitive function was assessed by a global cognition score, which included episodic memory, visuospatial abilities, calculation, orientation and attention.

## INTRODUCTION

In 2019, Medicare and Medicaid payments for patients with dementia was 2-22 times greater than patients without dementia. A total of 16 million Americans spent 18.6 billion hours of care for people with dementia; this care was valued at $244 billion (US). As a cause of ageing population, the number of people living with dementia will increase by 10 million per year worldwide, which will bring heavy economic costs and health burden [1]. Cognitive function tests are a key dementia detection method. Because dementia causes irreversible effects on quality of life and a strain on global health, it is imperative to identify modifiable risk factors for lower cognitive function to prevent or postpone dementia.

Considerable studies have found associations between sleep and cognitive function [2]. A recent meta-analysis revealed an inverted-U-shaped association between sleep duration and the risk of Alzheimer’s disease [3]**.** The study found that, compared with people with moderate sleep duration (MSD), both short sleep duration (SSD) and long sleep duration (LSD) were associated with a higher risk.

Experimental studies have shown associations between sleep, circadian rhythm, and neurodegenerative diseases, especially Alzheimer's disease [4, 5]. Under the guidance of this association, researchers have noted that an improvement in sleep duration might be a therapeutic strategy for dementia treatment or prevention [5–7]. Few studies have examined the effect of changes in sleep duration on cognition. Limited by sample sizes or methodology, the conclusions of all these studies were that increased or decreased sleep duration is associated with a lower cognitive function or a higher risk of dementia [8–10]. To date, no epidemiological or clinical study has demonstrated that improvement in sleep duration correlates with better cognitive function. The association between changes in sleep duration and cognition has yet to be examined among both middle-aged and older Chinese individuals.

Considering the “inverted-U-shaped association” mentioned above, we hypothesized that a change towards the optimal sleep duration would be associated with better cognitive function, while deviation from the optimal duration would be linked to lower cognitive function. To verify this hypothesis, a longitudinal cohort need to be used. Around 5% of the older adults have dementia. [1] Risk factors among the middle-aged are more relevant to cognitive change, than that among the older. [11] Here, we used a large-scale population-based prospective study, the China Health and Retirement Longitudinal Study (CHARLS), which focused on both middle-aged and older Chinese participants. The aim of our study was to (1) re-examine the inverted-U-shaped association between sleep duration and cognitive function among Chinese participants; (2) investigate the longitudinal association between baseline sleep duration and cognitive function over a period of time; and (3) study the association between changes in sleep duration and cognitive function.

## RESULTS

### Demographic and health characteristics of the study population in Wave 1

In Wave 1, the mean age of all participants was 59.1 ± 9.8 years; 47.3% of the participants were male, 76.5% were from rural areas, and 88.0% were married. With regard to education, 73.3% of the participants attended primary school and above. The mean ± SD nocturnal sleep duration was 6.4 ± 1.9 hours per night. At baseline, 62.5% of the participants were moderate sleepers (6-8 h), 29.2% were short sleepers (<6 h), and only 8.2% were long sleepers (>8 h); 0.3% of the participants used tranquilizers (Supplementary Figure 3 and Supplementary Table 8).

In Wave 1, the mean age of moderate sleepers was 2.9 years younger than that of short sleepers and 1.6 years younger than that of long sleepers (Table 1). Moderate sleepers were more likely to be male, live in urban areas, have better education, and be married. Moderate sleepers had a slightly higher body mass index (BMI) and a lower number of instrumental activities of daily living (IADLs), and they more often smoked and consumed alcohol. Long sleepers were more likely to suffer from depression. Short sleepers were more likely to have a history of hypertension, dyslipidaemia, heart disease and stroke, while moderate sleepers tended to be healthier (Table 1).

**Table 1:** Demographic and health characteristics of the study population in Wave 1^*^.

	**<6 h (n=4453)**	**6-8 h (n=9508)**	**>8 h (n=1255)**	**All (n=15216)**	***P*^a^**	***P*^b^**
Continuous variables					
Age	61.0 (10.0)	58.1 (9,5)	59.7 (10.7)	59.1 (9.8)	<0.001	<0.001
BMI	23.0 (3.6)	23.6 (3.6)	23.2 (3.6)	23.4 (3.7)	<0.001	<0.001
Number of IADLs	0.3 (0.9)	0.2 (0.6)	0.3 (0.9)	0.2 (0.7)	<0.001	<0.001
Categorical variables, n (%)					
Males	1889 (42.4)	4711 (49.6)	587 (46.9)	7187 (47.3)	<0.001	0.07
Education					<0.001	<0.001
Illiterate	1492 (33.5)	2153 (26.7)	415 (33.1)	4060 (26.7)		
Primary school	1872 (42.1)	3667 (38.6)	513 (40.9)	6052 (39.8)		
Middle school	732 (16.5)	2211 (23.3)	222 (17.7)	3165 (20.8)		
High school and above	354 (8.0)	1470 (15.5)	105 (8.4)	1929 (12.7)		
Marital status					<0.001	<0.001
Married	3742 (84.0)	8566 (90.1)	1085 (86.5)	13393 (88.0)		
Other status	711 (16.0)	959 (9.9)	170 (13.5)	1823 (12.0)		
Residential area					<0.001	<0.001
Urban	847 (19.1)	2522 (26.6)	205 (16.4)	3574 (23.5)		
Rural	3600 (81.0)	6975 (73.4)	1047 (83.6)	11622 (76.5)		
Depression	301 (6.8)	654 (6.9)	118 (9.5)	1073 (7.1)	0.80	0.001
Use of tranquilizers	25 (0.6)	20 (0.2)	4 (0.0)	49 (0.3)	<0.001	0.44
Current smoking	1660 (37.2)	3799 (40.0)	467 (37.2)	5926 (39.0)	0.001	0.06
Current alcohol consumption	1040 (23.4)	2467 (26.0)	276 (22.0)	3783 (24.9)	0.003	0.003
Hypertension	1187 (26.7)	2193 (22.9)	329 (25.5)	3688 (24.2)	<0.001	0.04
Dyslipidaemia	447 (10.0)	848 (8.9)	103 (8.2)	1397 (9.2)	0.03	0.41
History of heart disease	680 (15.3)	1041 (11.1)	118 (9.4)	1839 (12.1)	<0.001	0.10
History of stroke	122(2.7)	163(1.7)	27(2.2)	312 (2.1)	<0.001	0.27

^*^Three groups categorized by sleep duration in Wave 1.

^a^ *P* value for participants who slept <6 hours in Wave 1 compared with those who slept 6-8 hours.

^b^ *P* value for participants who slept >8 hours in Wave 1 compared with those who slept 6-8 hours.

### Associations between baseline sleep duration and cognitive function

### Baseline sleep duration and cognitive function in one wave

Supplementary Figure 1 showed the associations between sleep duration in Wave 1 and global cognition in Wave1. Supplementary Figure 1A, 1B visualized the result of generalized additive model (GAM) for global cognition score in Wave 1. The EDF values indicated a non-linear fit. The plots showed an inverted-U shaped association of sleep duration and global cognition score in both univariable and multivariable GAM models. The highest global cognition scores in Wave 1 were observed among those sleeping 6-8 hours per night in Wave 1 (Supplementary Figure 1).

The results remained unchanged when examining the association between sleep duration in Wave 1 and global cognition in Wave 3 (Supplementary Figure 2).

### Baseline sleep duration and longitudinal cognitive function in three waves

Short sleepers in Wave 1 had lower global cognition scores (β=-0.94, *P*<0.001 for model 1 and β=-0.53, *P*<0.001 for model 2) than moderate sleepers (Table 2) as well as lower scores in the specific domains episodic memory, figure drawing, and Telephone Interview of Cognitive Status (TICS) (Supplementary Table 1). Expressed in age equivalents and compared with the moderate sleep-duration group, the effect of short sleep was equivalent to being 9 (model 1) or 5 (model 2) years older according to the global cognition score (data not shown). Longer sleepers in Wave 1 had lower global cognition scores (β=-1.05, *P*<0.001 for model 1and β=-0.59, *P*<0.001 for model 2) than participants with a MSD and had lower scores in all three domains. Expressed in age equivalents and compared with the moderate sleep-duration group, the effect of long sleep time was equivalent to being 11 (model 1) or 6 (model 2) years older according to the global cognition score.

**Table 2:** Associations between baseline sleep duration and longitudinal global cognition according to generalized estimating equations (GEE).

	**Model 1**	**Model 2**	**Model 3**
Categorical trend			
SSD (<6 h)	-0.94 (-1.00, -0.88)^***,a^	-0.53 (-0.59, -0.47)^***^	
MSD (6-8 h)	Ref.	Ref.	
LSD (>8 h)	-1.05 (-1.16, -0.95)^***^	-0.59 (-0.68, -0.50)^***^	
SSD*time			0.02 (0.00, 0.04)
MSD*time			Ref.
LSD*time			-0.03 (-0.07, 0.01)
Linear trend for sleep duration ≤7 h			
Duration	0.48 (0.45, 0.51)^***^	0.28 (0.26, 0.30)^***^	
Duration*time			0.00 (-0.01, 0.01)
Linear trend for sleep duration≤7 h			
Duration	-0.47 (-0.49, -0.45)^***^	-0.28 (-0.32, -0.24)^***^	
Duration*time			-0.01 (-0.02, 0.01)

Abbreviations: SSD, short sleep duration; MSD, moderate sleep duration; LSD, long sleep duration.

^***^P<0.001

^a^Unstandardized beta coefficient (95% confidence interval).

Model 1: adjusted for age and sex.

Model 2: adjusted for Model 1+ education, marital status, residential area, depression, IADLs, use of tranquilizers, smoking, alcohol consumption, hypertension, dyslipidaemia, heart disease and stroke.

Model 3: adjusted for age and sex.

Linear trends were also significant. For participants who slept ≤ 7 h in Wave 1, a shorter sleep duration was associated with lower scores in global cognition (β=0.48, *P*<0.001 for model 1 and β=0.28, *P*<0.001 for model 2) and on all three tests. For participants who slept ≥ 7 h in Wave 1, higher sleep duration was associated with lower scores in global cognition (β=-0.47, *P*<0.001 for model 1 and β=-0.28, *P*<0.001 for model 2) and on all three tests.

The sleep-duration-by-time reaction was not significant (*P* > 0.10 for model 3), showing no significant association between sleep duration in Wave 1 and the rate of the reduction in global cognition score over a period of 4 years (Table 2).

### Associations between changes in sleep duration and cognitive function

### Participants who slept 6-8 h in Wave 1

In the analysis of the effect of changes in sleep duration from Wave 1 to Wave 2 (Table 3) and from Wave 1 to Wave 3 (Supplementary Table 3), among moderate sleepers (6-8 h) in Wave 1, we observed an inverted U-shaped association. Sleep durations that increased (β=-1.30, *P*<0.001 for model 1, β=-0.83, *P*<0.001 for model 2 and β=-0.63, *P*<0.001 for model 3) or decreased (β=-0.87, *P*<0.001 for model 1, β=-0.52, *P*<0.001 for model 2 and β=-0.37, *P*<0.001 for model 3) by ≥ 2 h over time were associated with lower global cognition scores than the those of the no-change group (Table 3). The effect of a ≥ 2 h change in Wave 2 was approximately equivalent to 9 years of cognitive ageing in model 1, model 2, and model 3. These associations were consistent across all three cognition tests for model 1, model 2 and model 3 (Supplementary Table 2).

**Table 3:** Associations between changes in sleep duration and global cognition in Wave 2 among participants who slept 6-8 hours at baseline^a^.

**Changes in sleep duration**					
	**Decreased by ≥ 2 h**	**Decreased by 0.5-1.5 h**	**No change**	**Increased by 0.5-1.5 h**	**Increased by ≥ 2 h**
Number of subjects	1487	1587	1690	1092	555
Mean (SD) score	7.79 (3.33)	8.76 (3.18)	8.80 (3.25)	8.43 (3.28)	7.25 (3.55)
Model 1	-0.87 (-0.98, -0.76) ^***,b^	-0.03 (-0.14, 0.07)	Ref.	-0.32 (-0.44, -0.20)^**^	-1.30 (-1.46, -1.15)^***^
Model 2	-0.52 (-0.62, -0.42)^***^	-0.02 (-0.12, 0.08)	Ref.	-0.14 (-0.25, -0.03)	-0.83 (-0.97, -0.69) ^***^
Model 3	-0.37 (-0.46, -0.28)^***^	-0.01 (-0.10, 0.08)	Ref..	-0.09 (-0.19, 0.01)	-0.63 (-0.76, -0.50)^***^

^a^ Using generalized linear models (GLMs).

^b^ Unstandardized beta coefficient (95% confidence interval).

^**^P<0.01, ^***^P<0.001.

Model 1: adjusted for age and sex.

Model 2: adjusted for Model 1+ education, marital status, residential area, depression, IADLs, use of tranquilizers, smoking, alcohol consumption, hypertension, dyslipidaemia, heart disease and stroke.

Model 3: adjusted for Model 2 + baseline global cognition score.

When analysing the effect of changes from Wave 1 to Wave 3, we found that the association between changes in sleep duration and global cognition remained unchanged (Supplementary Table 3). This association was found for all three tests for model 1. In models 2 and 3, the figure-drawing test exhibited no significant difference, while the episodic memory test and TICS test remained significant.

We also studied short sleepers in Wave 1, obtaining a similar result that a ≥ 2 h change in Wave 2 or Wave 3 was associated with lower global cognition scores. For long sleepers in Wave 1, the effect made no difference (data not shown).

### Participants who slept <6 h in Wave 1

The effect of sleep change on participants who slept < 6 h in Wave 1 was studied by subgroup analysis (Table 4). Compared with the “No-change” group, the “Excessive” group had lower global cognition in Wave 3 (β=-1.91, *P*<0.001 for model 1, β=-0.94, *P*<0.001 for model 2 and β=-0.53, *P*<0.05 for model 3). This result was found for all three tests. The “Benefit 1” group showed no significant difference. The “Benefit 2” group had a higher global cognition score (β=0.55, *P*<0.01 for model 1, β=0.54, *P*<0.01 for model 2 and β=0.38, *P*<0.05 for model 3). The effect of “Benefit 2” was approximately equivalent to 4-10 years of ageing. These results were found for figure drawing and TICS, while episodic memory exhibited no significant difference (Supplementary Table 4).

**Table 4:** Associations between change in sleep duration and global cognition in Wave 3 among participants who slept <6 hours or >8 hours at baseline^a^.

**Type of change in sleep duration ^c^**				
	**Excessive**	**No change**	**Benefit 1**	**Benefit 2**
SSD (<6 h) in Wave 1				
Number of subjects	224	1231	937	602
Mean (SD) score	7.23 (4.42)	9.39 (4.36)	9.27 (4.23)	10.35 (4.03)
Model 1	-1.91 (-2.21, -1.61)^***,b^	Ref.	-0.31 (-0.47, -0.13)	0.55 (0.36, 0.76)^**^
Model 2	-0.94 (-1.20, -0.94)^***^	Ref.	-0.22 (-0.37, -0.06)	0.54 (0.37, 0.71)^**^
Model 3	-0.53 (-0.76, -0.30)^*^	Ref.	-0.21 (-0.35, -0.07)	0.38 (0.22, 0.53)^*^
LSD (>8 h) in Wave 1				
Number of subjects	232	64	217	328
Mean (SD) score	8.19 (4.58)	8.77 (4.22)	9.47 (4.32)	10.47 (4.24)
Model 1	-0.68 (-1.26, -0.10)	Ref.	0.22 (-0.33, 0.76)	0.80 (0.26, 1.34)
Model 2	-1.38 (-1.90, -0.86)^**^	Ref.	-0.72 (-1.21, -0.20)	-0.34 (-0.86, 0.18)
Model 3	-1.17 (-1.62, -0.72)^**^	Ref.	-0.72 (-1.17, -0.27)	-0.43 (-0.89, 0.04)

Abbreviations: SSD, short sleep duration; MSD, moderate sleep duration; LSD, long sleep duration.

^c^ Type of change was decided by patterns of sleep duration in Wave 2 and Wave 3

For SSD in Wave 1: Excessive, as long as there was one wave with LSD; No change, maintained an SSD in two waves; Benefit 1, maintained an SSD in one wave and had an MSD in another; Benefit 2, MSD in two waves.

For LSD in Wave 1: Excessive, as long as there was one wave with an SSD; No change, maintained an LSD in the two waves; Benefit 1, maintained an LSD in one wave and had an MSD in another; Benefit 2, MSD in two waves.

^a^ Using generalized linear models (GLMs).

^b^ Unstandardized beta coefficient (95% confidence interval).

^*^P<0.05, ^**^P<0.01, ^***^P<0.001.

Model 1: adjusted for age and sex.

Model 2: adjusted for Model 1+ education, marital status, residential area, depression, IADLs, use of tranquilizers, smoking, alcohol consumption, hypertension, dyslipidaemia, heart disease and stroke.

Model 3: adjusted for Model 2 + baseline global cognition score.

### Participants who slept >8 h in Wave 1

The effect of sleep change on participants who slept > 8 h in Wave 1 is also shown in Table 4. Compared to the “No-change” group, the “Excessive” group had lower global cognition in Wave 3 (β=-1.38, *P*<0.01 for model 2 and β=-1.17, *P*<0.01 for model 3). This result was found for episodic memory. Participants in the “Benefit 1” group and the “Benefit 2” group had higher global cognition scores. However, there was no significant difference after adjusting for confounders.

## DISCUSSION

### Synopsis of findings

To date, this was the largest and most recent study examining the longitudinal association between self-reported sleep duration and cognitive function [3]. Using generalized estimation equations (GEE), our study indicated an inverted-U-shaped association between baseline sleep duration and global cognition over a period of 4 years among Chinese participants. The affected domains included episodic memory, figure drawing and TICS. However, baseline sleep duration did not increase the rates of cognitive decline.

For moderate sleepers, a ≥ 2 h change in sleep duration was significantly associated with lower global cognition and lower scores on all three tests. Relative to those whose sleep duration remained unchanged, a move from SSD to LSD was associated with lower global cognition, including the scores on all three tests, and a move from LSD to SSD was associated with lower episodic memory scores. For short sleepers, a consistent change to MSD was associated with high global cognition scores, equivalent to 4-10 years of cognitive ageing. The improved domains were figure drawing and TICS.

A change from LSD to MSD had no significant effect. However, only 8.2% of the participants were in the LSD group at baseline, and 0.6% of the participants remained in the LSD group from Wave 1 to Wave 3.

### Comparison with other studies

The most important finding of our study was that we challenged the previous ideas that increased or decreased sleep duration would lead to lower cognition. To date, a total of nine studies have examined the effect of changes in sleep duration on cognitive function or the risk of dementia. Five studies have linked increased sleep duration to lower cognition or a higher risk of dementia [8, 10, 12–14]; one study linked decreased sleep duration to a higher risk of Alzheimer's disease [15], and one study found no association [16]. Two studies reported that both increases and decreases in sleep duration were associated with lower cognition [9, 17]. According to the previous conclusion, if a person slept <6 h at baseline, and later slept 6-8 h, they would suffer a bad outcome, for their sleep duration had “increased”. Reversely, for long sleepers, a change to 6-8 h was also bad. To date, the three most important prospective studies regarding interventions to reduce the risk of cognitive decline or dementia did not include sleep duration [18]. “Sleep” has not yet been mentioned in the prevention tips suggested by the Alzheimer’s Association. Our new association provides evidence for intervention trials in the future.

The following reasons could explain why the conclusions of previous studies differed from ours. First, for moderate sleepers, our finding was in accordance with the previous study that a specific degree of change was harmful to cognition. Second, the effect of excessive change, showed by the “reverse group” in Table 4, was very strong. For short sleepers, the positive effect of change to MSD was obscured by the negative effect of excessive change to LSD. For long sleepers, the negative effect of a ≥ 2-hour decrease in sleep duration was caused by the change from LSD to SSD, while the change from LSD to MSD was not associated with worse cognition.

Table 5 shows studies focused on changes in sleep duration based on subgroup analysis. Based on the sleep duration at baseline, participants were divided into 3 groups: SSD, MSD, and LSD. The participants in the Chinese Longitudinal Healthy Longevity Survey had a mean age of 80 years, and 30% of them died during a 3-year follow-up. Age might have prevented the researchers from finding an association. Furthermore, the sleep pattern of their participants differed widely from those of participants in other studies; more (11.5% vs. <1%) of their participants remained in the LSD group [14]. The sleep duration in the Ohsaki Cohort 2006 study was recorded in 1994 and 2006. The sleep duration of humans declines as age increases [19]. The reduction in sleep duration caused by the long follow-up time would impact their results (Supplementary Table 7) [13]. The Whitehall Study had the relatively best study design; nonetheless, they did not obtain significant findings among people with SSD or have enough samples of individuals with LSD [9]. Remarkably, the findings in the 3 studies listed in Table 5 were not contradictory to our findings. These studies did not reach the level of significance of our findings and repeated the previous conclusion that increased or decreased sleep duration would cause lower cognition. Last but not least, in our study, a change from LSD to MSD had no effect. The Chinese Longitudinal Healthy Longevity Survey reported that a change from LSD to MSD could decrease the risk of dementia, which could be supplementary to our results.

**Table 5:** Summary of studies that linked changes in sleep duration with cognitive function or dementia using subgroup analysis.

**References**	**Participants**	**Measures of sleep duration**	**Cognitive tests and outcomes**	**Significant findings for SSD group**	**Significant findings for MSD group**	**Significant findings for LSD group**
Q. Zhu 2020	5419 Chinese participants aged 70-90 y from the Chinese Longitudinal Healthy Longevity Survey	Self-reported total hours of sleep in 2008 and 2011; MMSE at baseline and 2011	-Two MMSE tests at baseline and 3 y follow-up-MCI Criteria: follow-up MMSE score <18 for no education, MMSE<21 for primary education, MMSE<25 or middle education	-Change to LSD was associated with a higher risk of MCI, OR (1.41, 3.39)	-Change to LSD was associated with a higher risk of MCI, OR (1.02, 2.09)	-Change to MSD was associated with a lower risk of MCI, OR (0.45, 0.93)
Y. Lu 2018	7422 Japanese participants aged ≥ 65 y from the Ohsaki Cohort 2006 study	Self-reported total hours of sleep in 1994 and 2006	-One Dementia Scale test during 2007 and 2012-Incident dementia Criteria: dementia Scale rank ≥ 2	None	-Change to LSD was associated with higher risk of incident dementia, OR (1.13, 1.72)	None
J.E. Ferrie 2011	5431 British participants aged 45-69 from the Whitehall Study	Self-reported total hours of sleep in 1997 and 2002	-One MMSE test at the end-The score (ranged from 0 to 30)	None	-Change to SSD was associated with lower cognition, beta (-1.90, -0.49)-Change to LSD was associated with lower cognition, beta (-2.74, -0.72)	Insufficient sample

Abbreviations: MCI, mild cognitive impairment; SSD, short sleep duration; MSD, moderate sleep duration; LSD, long sleep duration.

Whether sleep duration increases the rate of cognitive decline is controversial [17, 20–25]. Some researchers have reported that a LSD at baseline could lead to a faster decline in cognition, while some reported no association. Using GEE, we did not find an association between baseline sleep duration and cognitive decline.

### Possible mechanisms explaining improvement in sleep duration and cognitive function

The circadian rhythm is composed of clock genes that exist in almost every cell of the human body and is part of the neuro-endocrine system. The circadian rhythm provides humans with the ability to adapt to the earth rotation every 24 hours. There is a bidirectional relationship between sleep duration, circadian rhythm and cognitive function [6]. Short or long sleep duration can disrupt the circadian rhythm [5]. Numerous studies have pointed to short or long sleep duration and disruption of circadian rhythm as a risk factor for neurodegenerative diseases, including dementia. [4, 26] The sleep-related circadian rhythm was reported to alter factors associated with neurodegeneration, including Aβ dynamics, clearance of toxic proteins, synaptic homeostasis, blood brain barrier and neuroinflammation. [27] Clinical studies have reported that restoration of circadian rhythm might reduce the deterioration of human cognitive function by using controlled light exposure or injection of melatonin, an endocrine hormone associated with circadian rhythm [28–31]. In an animal study, scientists improved the sleep duration of mice carrying the Huntington’s Disease (HD) mutation, slowing the cognitive decline of mice and reversing the dysregulation of their circadian rhythm. [32, 33] These studies suggested that a change from LSD to MSD might restore the circadian rhythm and thus lead to improved cognitive function in our participants. Conversely, excessive change and deviation from MSD correlated with disruption of the circadian rhythm, which was associated with worse cognitive function.

Furthermore, other mechanisms were discovered to explain the cross-sectional association between sleep duration and cognitive function, such as inflammatory markers, sleep apnoea and sleep fragmentation. Nonetheless, whether changes in sleep duration is associated with the levels of these mediators is not clear.

### Strengths and limitations

A strength of our study was its large and nationally representative sample of middle-aged and older Chinese individuals who were enrolled in a prospective and up- to-date study. The robustness of the CHARLS survey allowed us to adjust for multiple confounders.

Our study also had several limitations. First, measurement of sleep duration was self-reported, which could be influenced by recall bias. *C.L. Jackson 2018* studied the differences between self-reported and objectively measured sleep duration among several ethnic groups. Compared with white (73 min, 95% CI: 67-79), black (54 min, 95% CI: 42-65) and Hispanic (67 min, 95% CI: 56-78) individuals, Chinese individuals had the lowest bias (49 min, 95% CI: 37-61) [34]. Several cohorts have found an association between self-reported sleep duration and unhealthy outcomes, making our research convincing [35]. Most importantly, self-reported measurement is cheap and practicable, increasing the applicability of our results for health education and general research. Second, sleep-disordered breathing (SDB), which was not assessed in our study, has been found to be associated with lower cognition. [6, 36, 37]. A recent study assessed 5247 participants with in-home polysomnography and found no association between cognitive function and SDB assessments, including the Epworth Sleepiness Scale and Respiratory Event Index [7]. Third, 15.9% of participants in Wave 1 were lost to follow-up in Wave 2, and 19.4% were lost to follow-up in Wave 3. This loss might have affected our results.

### For health education

For short sleepers, a consistent change to moderate sleep duration correlated with better cognition. For long sleepers, there was no need to reduce sleep duration. Excessive changes or deviation from a moderate sleep duration was associated with lower cognition.

## MATERIALS AND METHODS

### Study sample

CHARLS is a longitudinal cohort study aimed to collect nationally representative sample of Chinese residents. The subjects of the study were Chinese citizens aged 45 years and above. The Wave 1 of CHARLS selected approximately 17000 individuals randomly from 150 counties/districts and 450 villages/resident committees. [38] Baseline data (Wave 1) were collected between June 2011 and March 2012. In addition, the study was conducted and followed up every two years.

**Figure 1:**
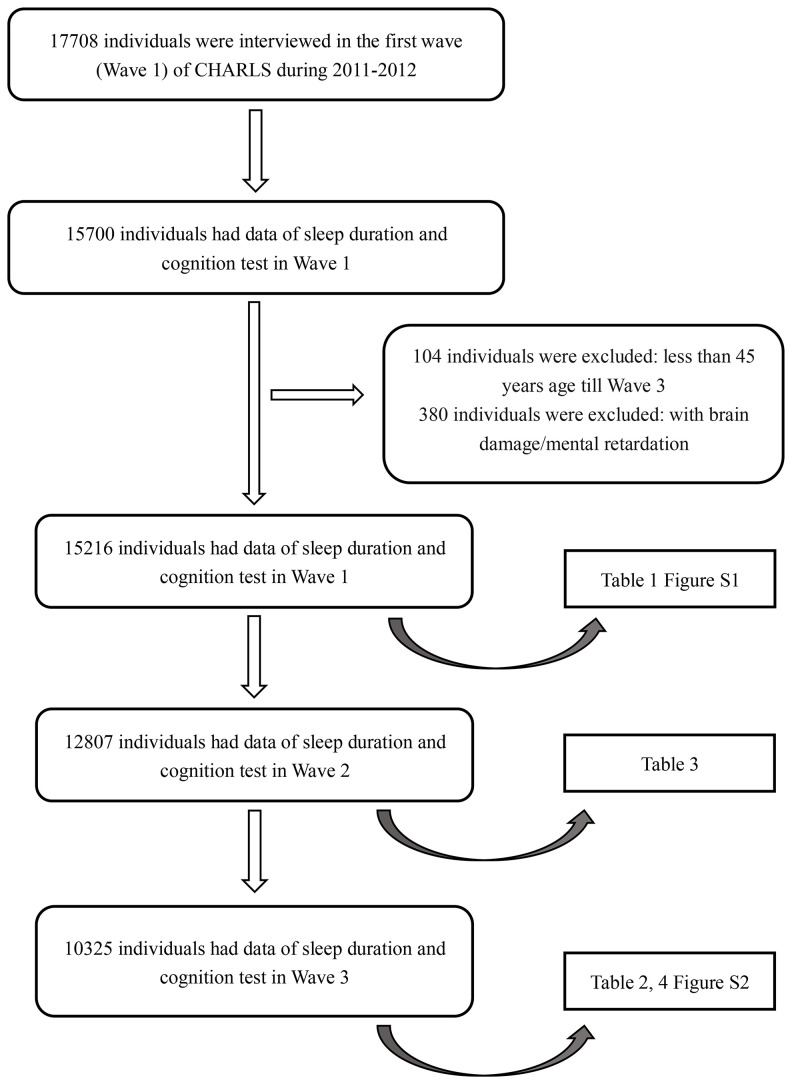
**Flow chart of sample selection and the exclusion criteria.**

In the CHARLS, a total of 15700 participants had data on sleep duration and cognitive tests in 2011 (Wave 1). 104 individuals under 45 years old in 2015 were excluded. A total of 380 individuals with a history of brain damage or mental retardation were excluded. Among the remaining 15216 participants, 12807 (84.2%) were followed up and had complete data on sleep duration and cognitive tests in 2013 (Wave 2). A total of 10325 (80.6%) participants were followed up and had related data in 2015 (Wave 3). The selection diagram and criteria for exclusion were provided in Figure 1.

### Ethics statement

Each participant included in this study signed a written informed consent form before taking the survey. Ethics approval for the data collection in the CHARLS was obtained from the Biomedical Ethics Review Committee of Peking University (IRB00001052-11015). We confirm that all methods were performed in accordance with the relevant guidelines and regulations.

### Assessment of cognitive function

The cognitive dimensions of memory, visuospatial abilities, orientation, attention and calculation were assessed through three tests: episodic memory, figure drawing, and TICS. Serving as the primary outcome, the global cognition score was the sum of the three test scores. The global cognition score could range from 0 to 21.

In the episodic memory test, the individuals were asked to recall words immediately (immediate recall) and 5 min later (delayed recall) after interviewers read 10 Chinese nouns to them. The episodic memory score was the average score of the immediate recall and delayed recall tests and could range from 0 to 10 [39]. This test was used to assess memory.

In the figure-drawing test, the individuals were shown a picture and asked to redraw it. Those who succeeded in drawing the figure received a score of 1. If they failed, they received a score of 0. This test was used to assess visuospatial abilities.

The TICS test was based on selected questions from the TICS battery, a well-established measure of one’s ability related to orientation, attention and calculation. In this test, the participants were asked to repeatedly subtract 7 from 100 and to identify the date, season, and day of the week. The TICS scores could range from 0 to 10 [40].

### Assessment of sleep duration

Nocturnal sleep duration was assessed using the following questions: “During the past month, how many hours of actual sleep did you get at night (average hours for one night)”? The data were accurate to 0.5 h.

### Potential confounders

The potential confounders included age, sex, education, marital status, residential area, cigarette smoking, alcohol consumption, depression, IADLs, use of tranquilizers, and comorbidities.

1293 individuals (13% of all individuals) lost BMI data in Wave 1. Considering the large proportion of missing data, we didn’t adjust for BMI in the main text. We included BMI as a confounder in Supplementary Table 5, as a sensitivity analysis. IADLs can range from 0 to 5 and reflect functional status [41]. Depression was classified as “yes” and “no”, using the 10-item Center for Epidemiologic Studies Short Depression Scale (CES-D-10). This score can range from 0 to 30, and the cut-off point for depression was 12 [42]. The comorbidities included hypertension, dyslipidaemia, history of stroke, and history of heart disease [43].

### Statistical analysis

Demographic characteristics are shown as the mean ± SD (standard deviation) or frequency (percentage). Associations between participants’ characteristics and baseline sleep duration were examined using ANOVA or the Pearson chi-square test.

We categorized sleep duration into three groups: SSD, <6 hours; MSD, 6-8 hours; and LSD, >8 hours. Nearly all previous studies chose 6 hours as the lower cut-off point. Because the mean sleep duration of our participants was 6.4 hours and the optimal sleep duration for the participants for global cognition was approximately 7 hours (Supplementary Figure 1, 2), we chose 8 hours as the upper cut-off point.

GEE was used to study the longitudinal association between baseline sleep duration and cognitive function over a period of 4 years. GEE accounted for between-participant variation and within-participant correlation of repeated outcomes. Time was defined as a continuous variable, measured as years from baseline. In model 1 and model 2, we calculated the estimates of sleep duration to examine the association between baseline sleep duration and cognition. In model 3, we calculated the estimates of the interaction of time and sleep duration to study the rate of cognitive decline [21, 44].

To analyse associations between changes in sleep duration and cognition, we used generalized linear models (GLMs) after adjustment for potential confounders.

As discussed above, the global cognition score was the primary outcome. Associations with the three cognition tests included in the global cognition score were examined in complementary analyses and were shown in the supplementary material.

As a sensitivity analysis, we further adjusted for BMI and day-time sleepiness in Supplementary Table 5, 6.

All statistical analyses were performed by R-3.4.3 and SAS version 9.4 (SAS Institute Inc., Cary, NC, USA).

### Ethics statement

Each participant included in this study signed a written informed consent form before taking the survey. Ethics approval for the data collection in the CHARLS was obtained from the Biomedical Ethics Review Committee of Peking University (IRB00001052-11015). We confirm that all methods were performed in accordance with the relevant guidelines and regulations.

## Supplementary Material

Supplementary Figures

Supplementary Tables
