# Supplementary material for: Improvement in sleep duration was associated with higher cognitive function: a new association
Source: Aging (Albany NY). 2020 Oct 20;12(20):20623–44. doi: 10.18632/aging.103948 (PMC7655193; doi:10.18632/aging.103948)
Supplement: Supplementary Figures [file aging-12-103948-s001..pdf]

SUPPLEMENTARY FIGURES

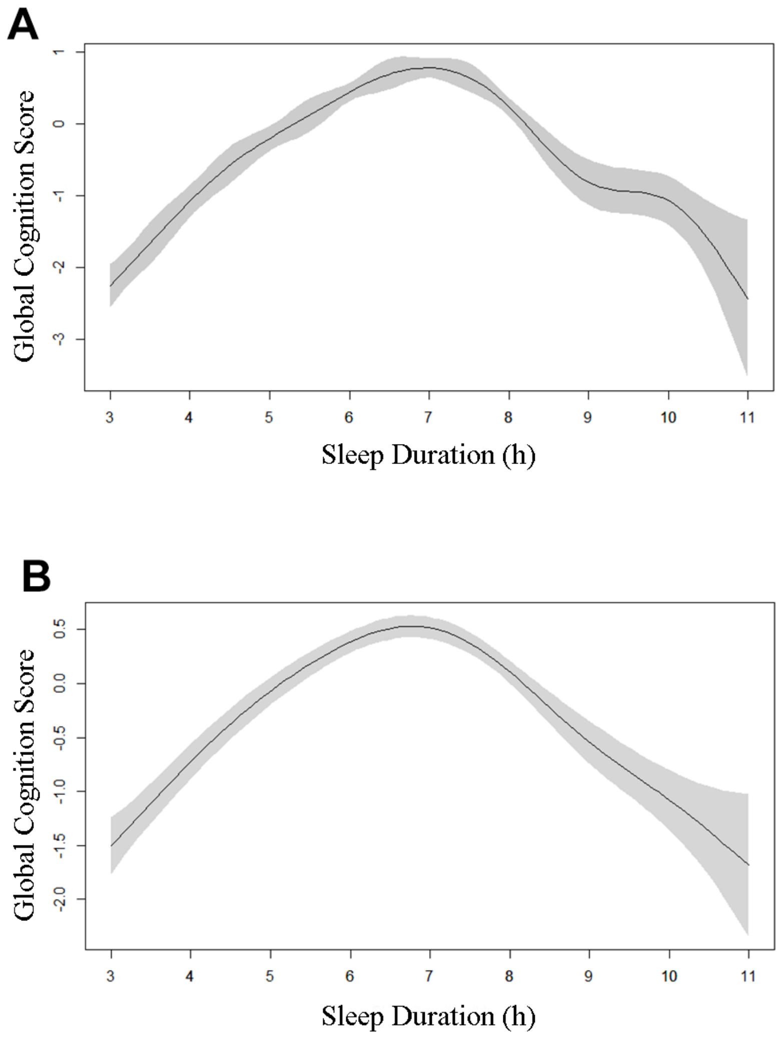

Supplementary Figure 1. Plots of the estimated smooth function of sleep duration in Wave 1 with 95% confidence intervals for the GAM when the response variable was global cognition in Wave 1. (**A**) Model 0 showed a univariate smooth function of sleep duration (EDF = 7.44, *P* < 0.001). (**B**) Model 1 presented a multivariable smooth function of sleep duration, adjusted for age and sex (EDF = 4.74, *P* < 0.001).

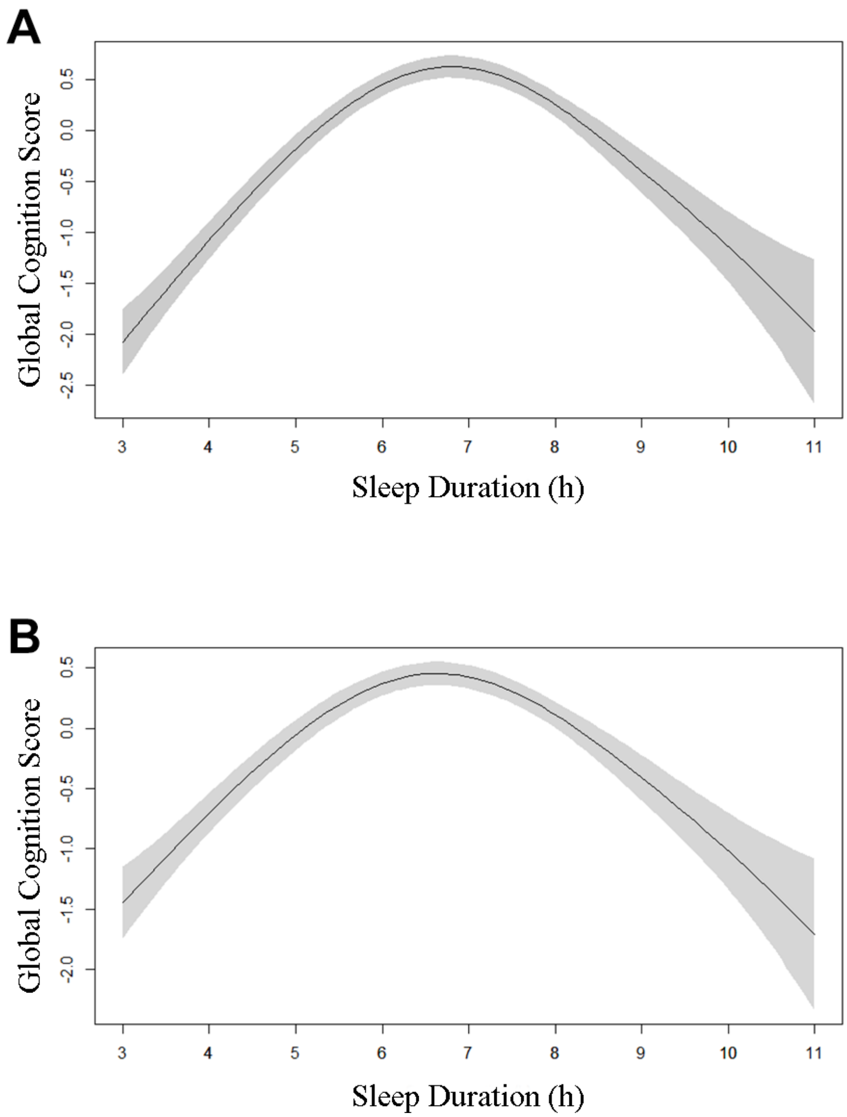

Supplementary Figure 2. Plots of estimated smooth function of sleep duration in Wave 1 with 95% confidence intervals for the GAM when the response variable was global cognition in Wave 3. (**A**) Model 0 showed a univariate smooth function of sleep duration (EDF = 3.71, P < 0.001). (**B**) Model 1 presented a multivariable smooth function of sleep duration, adjusted for age and sex (EDF = 4.58, P < 0.001).

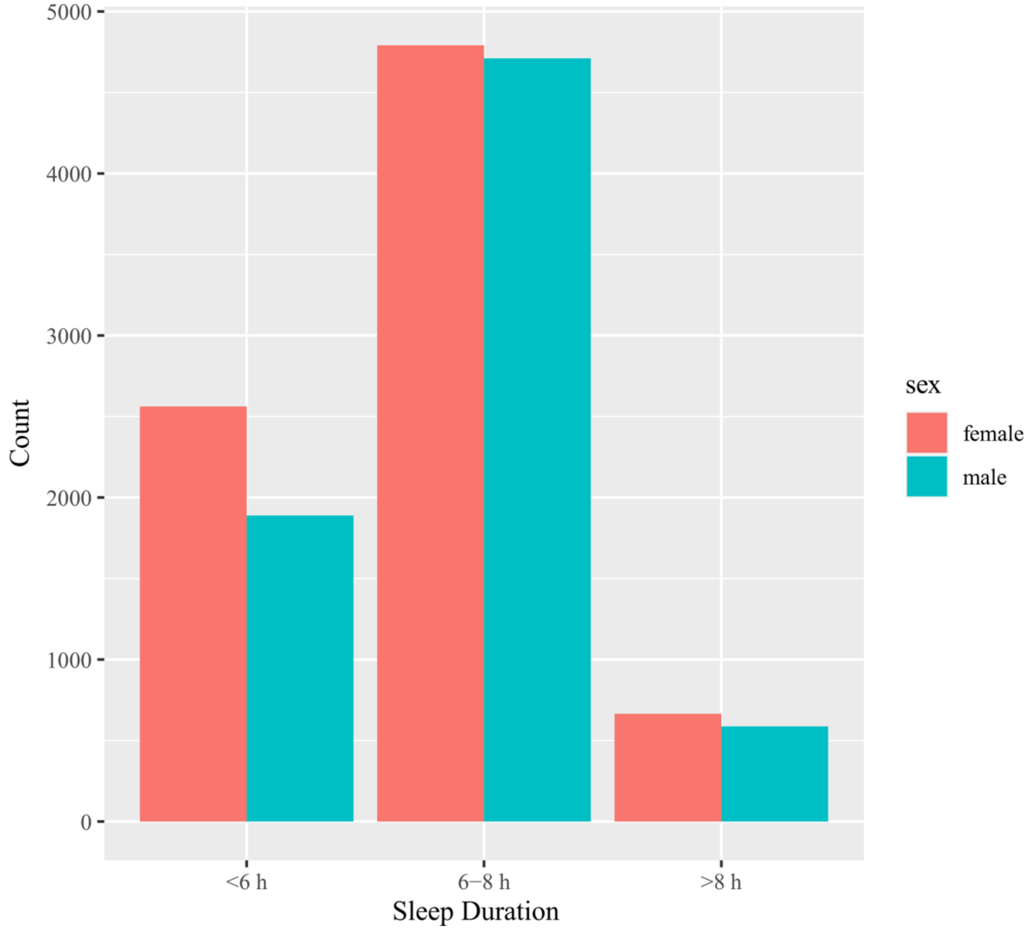

Supplementary Figure 3. Sex differences in sleep duration in Wave 1.
