# Supplementary material for: Improvement in sleep duration was associated with higher cognitive function: a new association
Source: Aging (Albany NY). 2020 Oct 20;12(20):20623–44. doi: 10.18632/aging.103948 (PMC7655193; doi:10.18632/aging.103948)
Supplement: Supplementary Tables [file aging-12-103948-s002..pdf]

SUPPLEMENTARY TABLES

Supplementary Table 1. Associations between baseline sleep duration and longitudinal cognitive function according to generalized estimating equations (GEE).

Episodic Memory			
	**Model 1 β(SE)**	**Model 2 β(SE)**	**Model 3 β(SE)**
Categorical trend			
SSD (<6 h)	-0.29 (0.03) ***	-0.16 (0.03) ***	
MSD (6-8 h)	Ref.	Ref.	
LSD (>8 h)	-0.32 (0.05) ***	-0.16 (0.04) ***	
SSD*time			-0.02 (0.01)
MSD*time			Ref.
LSD*time			-0.03 (0.01)
Linear trend for sleep duration ≤ 7 h			
Duration			0.15 (0.01) ***
Duration*time			0.00 (0.00)
Linear trend for sleep duration ≥ 7 h			
Duration			-0.16 (0.02) ***
Duration*time			-0.01 (0.01)

Figure drawing			
	Model 1 β(SE)	Model 2 β(SE)	Model 3 β(SE)
Categorical trend			
SSD (<6 h)	-0.08 (0.01) ***	-0.05 (0.01) ***	
MSD (6-8 h)	Ref.	Ref.	
LSD (>8 h)	-0.09 (0.01) ***	-0.04 (0.01) ***	
SSD*time			-0.01 (0.00)
MSD*time			Ref.
LSD*time			0.00 (0.00)
Linear trend for sleep duration ≤ 7 h			
Duration	0.04 (0.00) ***	0.02 (0.00) ***	
Duration*time			0.00 (0.00)
Linear trend for sleep duration ≥ 7 h			
Duration	-0.04 (0.01) ***	-0.01 (0.00) ***	
Duration*time			0.00 (0.00)

TICS			
	Model 1 β(SE)	Model 2 β(SE)	Model 3 β(SE)
Categorical trend			
SSD (<6 h)	-0.56 (0.04) ***	-0.32 (0.04) ***	
MSD (6-8 h)	Ref.	Ref.	
LSD (>8 h)	-0.65 (0.07) ***	-0.39 (0.06) ***	
SSD*time			0.00 (0.02)
MSD*time			Ref.
LSD*time			0.00 (0.03)
Linear trend for sleep duration ≤ 7 h			
Duration	0.29 (0.02) ***	0.17 (0.01) ***	
Duration*time			0.00 (0.01)
Linear trend for sleep duration ≥ 7 h			
Duration	-0.28 (0.03) ***	-0.17 (0.03) ***	
Duration*time			0.00 (0.01)

Abbreviations: SSD, short sleep duration; MSD, moderate sleep duration; LSD, long sleep duration.
***P<0.001, β: unstandardized beta coefficient, SE: standard error.
Model 1: adjusted for age and sex.
Model 2: adjusted for Model 1+ education, marital status, residential area, depression, IADLs, use of tranquilizers, smoking, alcohol consumption, hypertension, dyslipidaemia, heart disease and stroke. Model 3: adjusted for age and sex.

Supplementary Table 2. Associations between changes in sleep duration and cognitive function in Wave 2 among participants who slept 6-8 h at baseline[a].

	Changes in sleep duration				
	Decreased by ≥ 2 h	Decreased by 0.5-1.5 h	No change	Increased by 0.5-1.5 h	Increased by ≥ 2 h
Episodic memory					
Mean (SD) score	3.55 (1.73)	3.88 (1.73)	3.92 (1.77)	3.73 (1.73)	3.19 (1.89)
Model 1	-0.35 (0.06) ***,b	-0.05 (0.06)	Ref.	-0.19 (0.06) **	-0.61 (0.08) ***
Model 2	-0.21 (0.56) ***	-0.05 (0.06)	Ref.	-0.13 (0.06) *	-0.42 (0.08) ***
Model3	-0.17 (0.06) ***	-0.04 (0.05)	Ref..	-0.10 (0.06)	-0.37 (0.08) ***
Figure drawing					
Mean (SD) score	0.65 (0.48)	0.75 (0.43)	0.75 (0.43)	0.71 (0.45)	0.62 (0.49)
Model 1	-0.08 (0.02) ***	0.00 (0.01)	Ref.	-0.03 (0.02)	-0.10 (0.02) ***
Model 2	-0.04 (0.01) **	0.00 (0.01)	Ref.	-0.01 (0.02)	-0.05 (0.02) *
Model3	-0.04 (0.01) **	0.00 (0.01)	Ref..	0.00 (0.02)	-0.05 (0.02) *
TICS					
Mean (SD) score	3.59 (2.05)	4.12 (1.97)	4.13 (1.99)	3.99 (2.03)	3.44 (2.13)
Model 1	-0.44 (0.07) ***	-0.02 (0.07)	Ref.	-0.10 (0.08)	-0.59 (0.10) ***
Model 2	-0.27 (0.07) ***	-0.02 (0.70)	Ref.	0.00 (0.07)	-0.36 (0.09) ***
Model3	-0.19 (0.06) **	0.02 (0.06)	Ref..	0.00 (0.07)	-0.27 (0.09) **

[a] Using generalized linear models (GLM).
[b] Unstandardized beta coefficient (standard error), for all such values.
*P<0.05, **P<0.01, ***P<0.001.
Model 1: adjusted for age and sex.
Model 2: adjusted for Model 1+ education, marital status, residential area, depression, IADLs, use of tranquilizers, smoking, alcohol consumption, hypertension, dyslipidaemia, heart disease and stroke.
Model 3: adjusted for Model 2 + baseline global cognition score.

Supplementary Table 3. Associations between change in sleep duration and cognitive function in Wave 3 among participants who slept 6-8 h at baseline[a].

	Changes in sleep duration				
	Decreased by ≥ 2 h	Decreased by 0.5-1.5 h	No change	Increased by 0.5-1.5 h	Increased by ≥ 2 h
Number of subjects	1417	1409	1732	1138	794
Global cognition					
Mean (SD) score	10.06 (4.25)	11.39 (3.91)	11.51 (3.92)	10.07 (4.10)	9.57 (4.31)
Model 1	-1.17 (0.14)***,b	-0.04 (0.13)	Ref.	-0.34 (0.14)*	-1.56 (0.17)***
Model 2	-0.70 (0.12)***	0.02 (0.12)	Ref.	-0.16 (0.13)	-0.89 (0.15)***
Model3	-0.43 (0.11)***	0.12 (0.11)	Ref.	-0.09 (0.11)	-0.54 (0.13)***
Episodic memory					
Mean (SD) score	3.25 (1.78)	3.63 (1.78)	3.66 (1.81)	3.56 (1.86)	3.04 (1.83)
Model 1	-0.35 (0.06) ***,b	-0.03 (0.06)	Ref.	-0.09 (0.07)	-0.44 (0.26) ***
Model 2	-0.19 (0.06) ***	0.00 (0.06)	Ref.	-0.02 (0.06)	-0.22 (0.07) **
Model3	-0.15 (0.06) **	0.01 (0.05)	Ref..	-0.03 (0.06)	-0.15 (0.07) *
Figure drawing					
Mean (SD) score	0.60 (0.49)	0.71 (0.45)	0.70 (0.46)	0.67 (0.47)	0.60 (0.69)
Model 1	-0.07 (0.02) ***	0.03 (0.02)	Ref.	-0.02 (0.02)	-0.06 (0.02) **
Model 2	-0.02 (0.02)	0.03 (0.01) *	Ref.	0.00 (0.02)	0.00 (0.02)
Model3	-0.02 (0.02)	0.03 (0.01) *	Ref..	0.00 (0.02)	0.00 (0.02)
TICS					
Mean (SD) score	6.21 (2.89)	7.05 (2.62)	7.15 (2.61)	6.84 (2.69)	5.93 (2.96)
Model 1	-0.75 (0.10) ***	-0.04 (0.09)	Ref.	-0.24 (0.10)	-1.05 (0.11) ***
Model 2	-0.48 (0.09) ***	0.00 (0.08)	Ref.	-0.14 (0.09)	-0.67 (0.11) ***
Model3	-0.31 (0.08) ***	0.07 (0.07)	Ref..	-0.07 (0.08)	-0.47 (0.10) ***

[a] Using generalized linear models (GLM).
[b] Unstandardized beta coefficient (standard error), for all such values.
*P<0.05, **P<0.01, ***P<0.001.
Model 1: adjusted for age and sex.
Model 2: adjusted for Model 1+ education, marital status, residential area, depression, IADLs, use of tranquilizers, smoking, alcohol consumption, hypertension, dyslipidaemia, heart disease and stroke.
Model 3: adjusted for Model 2 + baseline global cognition score

Supplementary Table 4. Associations between change in sleep duration and cognitive function in Wave 3 among participants who slept <6 h or >8 h at baseline[a].

	Type of change in sleep duration [c]			
	Excessive	No change	Benefit 1	Benefit 2
Episodic memory				
SSD (<6 h) in Wave 1				
Mean (SD) score	2.26 (1.73)	3.00 (1.79)	2.98 (1.84)	3.25 (1.78)
Model 1	-0.65 (0.12)***	Ref.	-0.09 (0.07)	0.13 (0.08)
Model 2	-0.38 (0.12) **	Ref.	-0.06 (0.07)	0.13 (0.08)
Model 3	-0.32 (0.11) **	Ref.	-0.08 (0.07)	0.10 (0.08)
LSD (>8 h) in Wave 1				
Mean (SD) score	2.60 (1.84)	3.07 (1.85)	3.05 (1.77)	3.35 (1.85)
Model 1	-0.05 (0.02)**	Ref.	-0.24 (0.23)	-0.18 (0.24)
Model 2	-0.87 (0.23) **	Ref.	-0.53 (0.23) *	-0.08 (0.07) *
Model 3	-0.69 (0.21) **	Ref.	-0.48 (0.22)	-0.52 (0.23) *
Figure drawing				
SSD (<6 h) in Wave 1				
Mean (SD) score	0.35 (0.48)	0.53 (0.50)	0.53 (0.50)	0.64 (0.48)
Model 1	-0.16 (0.03)***	Ref.	-0.03 (0.02)	0.05 (0.02)*
Model 2	-0.08 (0.03) *	Ref.	-0.02 (0.02)	0.05 (0.02)*
Model 3	-0.08 (0.03) *	Ref.	-0.01 (0.02)	0.05 (0.02)*
LSD (>8 h) in Wave 1				
Mean (SD) score	0.49 (0.50)	0.52 (0.50)	0.57 (0.50)	0.67 (0.47)
Model 1	-0.02 (0.06)	Ref.	0.02 (0.07)	0.08 (0.06)
Model 2	-0.06 (0.07)	Ref.	-0.08 (0.07)	-0.01 (0.06)
Model 3	-0.06 (0.06)	Ref.	-0.08 (0.07)	-0.04 (0.06)
TICS				
SSD (<6 h) in Wave 1				
Mean (SD) score	4.62 (3.00)	5.85 (2.98)	5.77 (3.05)	6.47 (2.78)
Model 1	-1.10 (0.21)***	Ref.	-0.19 (0.13)	0.37 (0.14)**
Model 2	-0.48 (0.19) *	Ref.	-0.14 (0.11)	0.36 (0.13)**
Model 3	-0.19 (0.17)	Ref.	-0.03 (0.10)	0.25 (0.11)*
LSD (>8 h) in Wave 1				
Mean (SD) score	5.10 (3.18)	5.19 (2.89)	5.86 (3.01)	6.45 (2.91)
Model 1	-0.02 (0.40)	Ref.	0.44 (0.39)	0.90 (0.39)*
Model 2	-0.45 (0.38)	Ref.	-0.10 (0.37)	0.25 (0.38)
Model 3	-0.52 (0.34)	Ref.	-0.19 (0.35)	0.09 (0.34)

Abbreviations: SSD, short sleep duration; MSD, moderate sleep duration; LSD, long sleep duration.

[c] Type of change was decided by patterns of sleep duration in Wave 2 and Wave 3

For SSD in Wave 1: Excessive, as long as there was one wave with LSD; No change, maintained a SSD in two waves; Benefit 1, maintained a SSD in one wave and had a MSD in another; Benefit 2, MSD in two waves.

For LSD in Wave 1: Excessive, as long as there was one wave with a SSD; No change, maintained a LSD in the two waves; Benefit 1, maintained a LSD in one wave and had a MSD in another; Benefit 2, MSD in two waves.

[a] Using generalized linear models (GLMs).

[b] Unstandardized beta coefficient (standard error).

*P<0.05, **P<0.01, ***P<0.001.

Model 1: adjusted for age and sex.

Model 2: adjusted for Model 1+ education, marital status, residential area, depression, IADLs, use of tranquilizers, smoking, alcohol consumption, hypertension, dyslipidaemia, heart disease and stroke.

Model 3: adjusted for Model 2 + baseline global cognition score

Supplementary Table 5. Associations between change in sleep duration and global cognition score in Wave 3 among participants who slept <6 h or >8 h at baseline[a].

	Type of change in sleep duration [c]			
	Excessive	No change	Benefit 1	Benefit 2
SSD (<6 h) in Wave 1				
Number of subjects	193	1080	817	535
Mean (SD) score	7.34 (4.30)	9.43 (4.30)	9.08 (4.38)	10.32 (4.01)
Model 2	-0.98 (-1.26, -0.70)***	Ref.	-0.40 (-0.56, -0.24) *	0.56 (0.38, 0.74) **
Model 3	-0.55 (-0.80, -0.30) *	Ref.	-0.37 (-0.51, -0.23)**	0.41 (0.25, 0.57) *
LSD (>8 h) in Wave 1				
Number of subjects	283	55	118	285
Mean (SD) score	8.87 (4.33)	7.70 (4.60)	8.85 (4.37)	10.28 (4.30)
Model 2	-1.22 (-1.76, -0.68) *	Ref.	-0.55 (-1.07, -0.02)	-0.15 (-0.70, 0.39)
Model 3	-1.03 (-1.50, -0.56) *	Ref.	-0.59 (-1.08, -0.10)	-0.20 (-0.70, -0.29)

Supplementary Table 6. Associations between change in sleep duration and global cognition score in Wave 3 among participants who slept <6 h or >8 h at baseline[a].

	Type of change in sleep duration [c]			
	Excessive	No change	Benefit 1	Benefit 2
SSD (<6 h) in Wave 1				
Number of subjects	224	1231	937	602
Mean (SD) score	7.23 (4.42)	9.39 (4.36)	9.27 (4.23)	10.35 (4.03)
Model 2	-1.02 (-0.75, -0.21)***	Ref.	-0.23 (-0.39, -0.08)	0.47 (0.29, 0.55)**
Model 3	-0.56 (-0.80, -0.33)*	Ref.	-0.22 (-0.36, -0.08)	0.37 (0.21, 0.53)*
LSD (>8 h) in Wave 1				
Number of subjects	232	64	217	328
Mean (SD) score	8.19 (4.58)	8.77 (4.22)	9.47 (4.32)	10.47 (4.24)
Model 2	-1.36 (-1.88, -0.83)*	Ref.	-0.72 (-1.22, -0.22)	-0.32 (-0.83, 0.22)
Model 3	-1.17 (-1.63, -0.71)*	Ref.	-0.73 (1.19, -0.24))	-0.42 (-0.88, 0.02)

As discussed in question 1, the main results were presented in Table 4. The associations in Tables 2, 3 were strong enough (P < 0.001) that they would not be weakened. We only analyzed participants enrolled in Table 4.
1293 individuals (13% of all individuals) lost BMI data in Wave 1. Considering the large proportion of missing data, we didn't adjust for BMI in the main text.
We have now included BMI as a confounder in the Supplementary Material (Table S5), as a sensitivity analysis. We deleted the missing data and used BMI as a confounder; and also found that "Excessive" group was associated with lower global cognition among both short and long sleepers, and "Benefit 2" group was associated with high global cognition among short sleepers. Thus, in the sensitivity analyses, the result remained same.

Supplementary Table 7. Associations between sex, follow-up time and longitudinal sleep duration according to generalized estimating equations (GEE).

Sleep duration	Gender[b] β (95%CI)	Age	Time β (95%CI)	Gender*time β (95%CI)
Model 1	-0.32 (-0.35, -0.29) ***	-0.02 (-0.01, -0.02) ***	-0.02 (-0.01, -0.02) ***	
Model 2	-0.21 (-0.17, 0.25) ***	-0.02 (-0.01, -0.02) ***	0.05 (0.03,0.06) **	-0.04 (-0.03, -0.05) ***

Model 1: The independent variables were age, sex, follow-up time
Model 2: The independent variables were age, sex, follow-up time, gender* follow-up time,
***P<0.001, **P<0.01
[b]Male was defined as 1 and female as 2.

Supplementary Table 8. Age of the population analyzed in Table 4 in 2015 (Wave 3).

	All	Male (n=4931)	Female (n=5387)
Mean (SD)	62.1 (8.9)	63.2 (8.7)	61.2 (8.9)
Median (IQR)	62 (13)	63 (12)	61 (14)

The age of all participants ranged from 45-97.
Kolmogorov-Smirnov tests for all groups were significant (P<0.01). Age did not exhibit a normal distribution. Kruskal-Wallis test showed a significant gender difference (P< 0.001). The mean age of females was 2 year younger than males.
Abbreviations: SD, standard deviation; IQR, interquartile range
